# Supplementary material for: EnzML: multi-label prediction of enzyme classes using InterPro signatures
Source: BMC Bioinformatics. 2012 Apr 25;13:61. doi: 10.1186/1471-2105-13-61 (PMC3483700; doi:10.1186/1471-2105-13-61)
Supplement: Addtional file 5 — The Java code to format the data files, evaluate and predict. The file enzml_java_code.tar.gz contains the Java code used to format database data to ARFF and XML formats, to execute cross and train-test (jackknife) evaluations and to record evaluation results to database. More information is included in the readme.txt file and the Javadoc files. The code can be used with a MySQL database. To use a different database software, other JDBC drivers might be required. [file 1471-2105-13-61-S5.gz › java_code/enzml2011/doc/uk/ac/ed/inf/taweka/weka/Arff.html]

Arff


---


|  |  |  |
| --- | --- | --- |
| **Overview** \| **Package** \| **Class** \| **Use** \| **Tree** \| **Deprecated** \| **Index** \| **Help** | |  |
| PREV CLASS   **NEXT CLASS** | **FRAMES**    **NO FRAMES**     **All Classes** |  |
| SUMMARY: NESTED \| FIELD \| CONSTR \| METHOD | DETAIL: FIELD \| CONSTR \| METHOD |  |


---


## uk.ac.ed.inf.taweka.weka Class Arff

```
java.lang.Object
  uk.ac.ed.inf.taweka.Initialised
      uk.ac.ed.inf.taweka.weka.Arff
```

**All Implemented Interfaces:**: Initialisable

**Direct Known Subclasses:**: MulanArff

---

``` public class Arff extends Initialised ```

Creates an ARFF file: checks file properties, loads data from database, fills
and writes to file the ARFF attributes, classes and instances, generates
auxiliary files if needed.

**Version:**
:   22 Apr 2010

**Author:**
:   Luna De Ferrari luna.deferrari-at-ed.ac.uk

---

| **Field Summary** | |
| --- | --- |
| `static java.lang.String` | `ARFF_TABLE`             The name of the table to save the arff file details to |
| `static java.lang.String` | `AUTHOR` |
| `static java.lang.String` | `TAWEKA_NAME` |
| `static java.lang.String` | `VERSION` |
| `static java.lang.String` | `WEB_PAGE` |

**Fields inherited from class uk.ac.ed.inf.taweka.Initialised**

`m_initialisationIsCorrect`


**Constructor Summary**

`Arff(java.lang.String dataPropsFilePath,
java.lang.String resultPropsFilePath)`


| **Method Summary** | |
| --- | --- |
| `int` | `generateAndWriteArffFile()`             The core of TaWeka: save properties to experiment table, load data from data database and save arff to file |
| `java.lang.String` | `getArffPath()` |
| `int` | `getArffRecordId()` |
| `ArffProperties` | `getDataArffProperties()` |
| `uk.ac.ed.inf.utils.database.DbManager` | `getDataDbManager()` |
| `java.lang.String` | `getDataPropsFilePath()` |
| `DataSetManager` | `getDataSetManager()` |
| `uk.ac.ed.inf.utils.database.DbConn` | `getDbConn()` |
| `uk.ac.ed.inf.utils.database.DbReader` | `getDbReader()` |
| `uk.ac.ed.inf.utils.database.DbManager` | `getResultsDbManager()` |
| `void` | `loadAndGenerateArff()` |
| `static void` | `main(java.lang.String[] args)` |
| `int` | `setArffRecordId(int arffRecordId)`             Sets the arff record id and returns it for further use. |
| `void` | `setDataSetManager()` |

**Methods inherited from class uk.ac.ed.inf.taweka.Initialised**

`initialisationIsCorrect, setInitialisationIsCorrect`

**Methods inherited from class java.lang.Object**

`equals, getClass, hashCode, notify, notifyAll, toString, wait, wait, wait`

**Field Detail**

### ARFF\_TABLE

```
public static final java.lang.String ARFF_TABLE
```

:   The name of the table to save the arff file details to

    **See Also:**: Constant Field Values

---


### AUTHOR

```
public static final java.lang.String AUTHOR
```

**See Also:**: Constant Field Values

---


### TAWEKA\_NAME

```
public static final java.lang.String TAWEKA_NAME
```

**See Also:**: Constant Field Values

---


### VERSION

```
public static final java.lang.String VERSION
```

**See Also:**: Constant Field Values

---


### WEB\_PAGE

```
public static final java.lang.String WEB_PAGE
```

**See Also:**: Constant Field Values


**Constructor Detail**

### Arff

```
public Arff(java.lang.String dataPropsFilePath,
            java.lang.String resultPropsFilePath)
```


**Method Detail**

### generateAndWriteArffFile

```
public int generateAndWriteArffFile()
```

:   The core of TaWeka: save properties to experiment table, load data from
    data database and save arff to file

---


### getArffPath

```
public java.lang.String getArffPath()
```

---


### getArffRecordId

```
public int getArffRecordId()
```

---


### getDataArffProperties

```
public ArffProperties getDataArffProperties()
```

---


### getDataDbManager

```
public uk.ac.ed.inf.utils.database.DbManager getDataDbManager()
```

---


### getDataPropsFilePath

```
public java.lang.String getDataPropsFilePath()
```

---


### getDataSetManager

```
public DataSetManager getDataSetManager()
```

---


### getDbConn

```
public uk.ac.ed.inf.utils.database.DbConn getDbConn()
```

---


### getDbReader

```
public uk.ac.ed.inf.utils.database.DbReader getDbReader()
```

---


### getResultsDbManager

```
public uk.ac.ed.inf.utils.database.DbManager getResultsDbManager()
```

---


### loadAndGenerateArff

```
public void loadAndGenerateArff()
```

---


### setArffRecordId

```
public int setArffRecordId(int arffRecordId)
```

:   Sets the arff record id and returns it for further use.

    :   **Parameters:**: `arffRecordId` - the table id for this arff file details **Returns:**: the table id for this arff file details

---


### setDataSetManager

```
public void setDataSetManager()
```

---


### main

```
public static void main(java.lang.String[] args)
```


---


|  |  |  |
| --- | --- | --- |
| **Overview** \| **Package** \| **Class** \| **Use** \| **Tree** \| **Deprecated** \| **Index** \| **Help** | |  |
| PREV CLASS   **NEXT CLASS** | **FRAMES**    **NO FRAMES**     **All Classes** |  |
| SUMMARY: NESTED \| FIELD \| CONSTR \| METHOD | DETAIL: FIELD \| CONSTR \| METHOD |  |


---
